# FBXO22 Promotes Growth and Metastasis and Inhibits Autophagy in Epithelial Ovarian Cancers *via* the MAPK/ERK Pathway

**DOI:** 10.3389/fphar.2021.778698

**Published:** 2021-12-07

**Authors:** Minle Li, Xue Zhao, Hongmei Yong, Bingqing Shang, Weihua Lou, You Wang, Jin Bai

**Affiliations:** ^1^ Cancer Institute, Xuzhou Medical University, Xuzhou, China; ^2^ Cancer Research Center, School of Medicine, Xiamen University, Xiamen, China; ^3^ Department of Oncology, The Affiliated Huai’an Hospital of Xuzhou Medical University and The Second People’s Hospital of Huai’an, Huai’an, China; ^4^ Department of Obstetrics and Gynecology, Renji Hospital, School of Medicine, Shanghai Jiao Tong University, Shanghai, China; ^5^ Shanghai Key Laboratory of Gynecologic Oncology, Shanghai, China; ^6^ State Key Laboratory of Oncogenes and Related Genes, Shanghai Cancer Institute, Renji Hospital, School of Medicine, Shanghai Jiao Tong University, Shanghai, China; ^7^ Center of Clinical Oncology, Affiliated Hospital of Xuzhou Medical University, Xuzhou, China

**Keywords:** FBXO22, epithelial ovarian cancers (EOCs), metastasis, autophagy, MAPK, ERK

## Abstract

E3 ubiquitin ligase F-box only protein 22 (FBXO22), which targets the key regulators of cellular activities for ubiquitylation and degradation, plays an important role in tumorigenesis and metastasis. However, the function of FBXO22 in epithelial ovarian cancers has not been reported. This study aims to explore the biological function of FBXO22 in epithelial ovarian cancers progression and metastasis and its specific regulation mechanism. Immunohistochemistry analysis of tissue microarray was performed to evaluate the expression of FBXO22 in epithelial ovarian cancers patients. The proliferative ability of epithelial ovarian cancers cells was examined by the CCK8. The metastasis ability was detected by the wound healing assay, migration and invasion assays. Western blot was used to verify the relationship between FBXO22 expression and mitogen-activated protein kinase related proteins. Autophagic flux was detected by electron microscopy, mRFP-GFP-LC3 adenovirus, lysosomal tracker and western blot. For *in vivo* experiments, the effect of FBXO22 on epithelial ovarian cancers resistance was observed in a xenograft tumor model and a metastatic mice model. We found that FBXO22 expression was significantly increased in epithelial ovarian cancers tissues and was closely correlated with clinical pathological factors. As a result, we found that FBXO22 promoted the growth and metastasis, as well as inhibited the autophagy flux. In addition, we identified that FBXO22 performed these functions *via* the MAPK/ERK pathway. Our results first reported the function of FBXO22 in epithelial ovarian cancer and the correlation between FBXO22 and autophagy, suggesting FBXO22 as a novel target of epithelial ovarian cancers assessment and treatment.

## Introduction

Ovarian cancer is the seventh most common cancer among women, and the age-standardized rates in developed and developing countries are approximately 9.4 and 5.0 per 100,000 people, respectively (Siegel et al., 2016; Reid et al., 2017). Despite developments in drug discovery and management, ovarian cancer remains the leading cause of death due to gynecological cancer (Miller et al., 2016). As many as 240,000 women worldwide are diagnosed with ovarian cancer, and nearly half of the women die every year (Reid et al., 2017). About 85–90% of all ovarian cancers are epithelial in origin, and around 70% of all epithelial ovarian cancers (EOCs) are high-grade serous (HGS) adenocarcinoma (McLachlan et al., 2016).

Early diagnosis increases the chance of recovery, but due to the nonspecific symptoms of the disease in the early stage, recovery is hindered. Advanced ovarian cancer is aggressive, has rapid growth and spread and chemotherapy/radiotherapy resistance, and recurs. At present, cytoreductive surgery is clinically adopted, along with chemotherapy using cisplatin and paclitaxel, but the multidrug resistance of ovarian cancer cells has severely reduced the long-term efficacy of these treatments; the 5-year survival rate is only 30% (Coleman et al., 2013). Therefore, issues concerning the in-depth exploration of the mechanism of the occurrence, development, invasion, and metastasis of ovarian cancer cells and in the search for effective targeted treatment approaches need to be resolved urgently.

Autophagy is a lysosome-dependent degradation pathway that widely occurs in eukaryotic cells. Under normal physiological conditions, autophagy facilitates the maintenance of cell homeostasis and promotes cell survival; however, excessive autophagy can cause cell death through a process called “autophagic cell death,” along with apoptosis and necrosis (Hippert et al., 2006). This “double-edged sword” effect in the regulation cell fate is manifested in the relationships among autophagy, tumorigenesis, and development, and depends on the different stages of disease progression, changes in the surrounding environment of cells, and different therapeutic interventions. Autophagy is a tumor suppressor mechanism, and decline in autophagy may be beneficial to tumor development. Tumor cells have a lower degree of autophagy ability than normal cells. The autophagy ability of normal cells first eliminates organelles (mainly mitochondria) damaged by chemical carcinogens, radiation, and oxidative stress to prevent genetic instability due to the damage caused by reactive oxygen species (ROS) to DNA, thereby hindering the proliferation of tumor cells. Autophagy can degrade the endoplasmic reticulum, Golgi apparatus, other organelles, and long-lived proteins, causing premalignant cells to be in a negative protein balance and inhibiting their uncontrolled proliferation (Hait et al., 2006).

The mitogen-activated protein kinase (MAPK) family, which includes ERK2⁄ERK1 (also known as p42⁄p44MAPK), p38 MAPK, ERK5, and c-Jun-NH2-terminal kinases (JNK1⁄2⁄3), plays a crucial role in nearly all cell functions (Ramos, 2008). Depending on duration, magnitude, and subcellular localization, ERK activation controls various cell responses, such as proliferation, migration, differentiation, and death (Murphy and Blenis, 2006). ERK increases cell survival by promoting the activities of antiapoptotic proteins, such as B cell lymphoma (Bcl)-2 and Bcl-XL. ERK-dependent autophagic activity is associated with the classical markers of autophagy, such as the induction of LC3, conversion of LC3-I to LC3-II (Ogier-Denis et al., 2000; Cheng et al., 2008; Sivaprasad and Basu, 2008; Oh and Lim, 2009), and induction of Beclin 1 (BECN1) (Cheng et al., 2008). Remarkably, Bcl-2 inhibits autophagy by binding to the BCL-2 homology 3 (BH3) domain of BECN1, which block the binding between BECN1 and vacuolar protein sorting 34 (Vps 34).

FBXO22 is a member of the F-box protein family (Tan et al., 2011; Dikopoltsev et al., 2014). The SCF F-box complex have pivotal roles in multiple cellular processes and tumorigenesis through ubiquitylation and subsequent degradation of target proteins (Wang et al., 2014), including proteins related to cell cycle progression, signal transduction, and transcription (Nakayama and Nakayama, 2006; Sarikas et al., 2008; Sarikas et al., 2011). Several biological functions of FBXO22 in tumorigenesis have been determined in recent years. According to the different degradation target proteins of the FBXO22 complex, it plays a corresponding function in cells (Cheng et al., 2020). FBXO22 promotes tumorigenesis by degrading nuclear PTEN (Ge et al., 2020), promotes lung cancer by mediating the polyubiquitination with the inactivation of LKB1 (Zhu et al., 2019) and Bach1 (Lignitto et al., 2019), and promotes the development of hepatocellular carcinoma by regulating the ubiquitination and degradation of p21 (Zhang et al., 2019). Our previous research shown that FBXO22 targets HDM2 for degradation and modulates breast cancer cell invasion and metastasis (Bai et al., 2019), and it suppresses metastasis in human renal cell carcinoma by inhibiting matrix metalloproteinase (MMP)-9-mediated migration and invasion and VEGF-mediated angiogenesis (Guo et al., 2019). Moreover, FBXO22 knockdown inhibits melanoma cell migration, invasion, and angiogenesis via the HIF-1α/VEGF pathway (Zheng et al., 2020). By contrast, the function of FBXO22 in epithelial ovarian cancers is unclear.

In this study, we demonstrated the expression of FBXO22 in patients. We investigated the biological functions of FBXO22 *in vitro* and *in vivo*. Furthermore, we explore the mechanism and prospected the potential and profound significance of FBXO22 in epithelial ovarian cancers assessment and treatment.

## Materials and Methods

### Patients and Samples

A tissue microarray (TMA) containing 251 EOCs tissues and 10 matched normal tissues were constructed by Jiangsu Cancer Biotherapy Institute (Xuzhou, China). The tissues, which were embedded in paraffin blocks, were collected from the Pathology Department of Affiliated Hospital of Xuzhou Medical University (Xuzhou, China). All the patients underwent definitive diagnosis of epithelial ovarian cancers and then radical surgery at the above hospital. Detailed clinical information of each specimen was recorded accurately and completely. All the tissue specimens were obtained from the patient, all of whom provided informed consent, and the use of human specimens was approved by the Review Board of the Affiliated Hospital of Xuzhou Medical College.

### Tissue Microarray Immunohistochemistry

TMA immunohistochemistry was implemented according to the streptavidin–peroxidase (Sp) method. A standard PV-9001 kit (Beijing Zhongshan Golden Bridge Biotechnology, Beijing, China) was used. Before immunostaining, TMA slides were dewaxed at 60°C for 2 h, then deparaffinized with xylene and hydrated with graded ethanol and distilled water. Endogenous peroxidases were inhibited with 3% H_2_O_2_ for 30 min. Antigen retrieval was performed by heating the TMA slides immersed in a retrieval solution (10 mM sodium citrate buffer, pH 6.0) at 95°C for 30 min in a pressure boiler. After 1 h of blocking with 5% normal goat serum, the sections were incubated with polyclonal rabbit anti-FBXO22 antibody (1:100 dilution, Proteintech) overnight at 4 °C. The slides were immersed in a response enhancer for 20 min and biotin-labeled secondary antibody (PV-9001 kit, Beijing Zhongshan Golden Bridge Biotechnology, Beijing, China) for 20 min at room temperature and then with avidin–peroxidase reagent and 3,3′-diaminobenzidine (Beijing Zhongshan Golden Bridge Biotechnology, Beijing, China) substrate. After hematoxylin counterstaining and dehydration, the sections were sealed with cover slips.

### Evaluation of Immunostaining

Two pathologists separately examined the TMAs under blinded experimental conditions. The staining scores of FBXO22 were evaluated according to the intensity and percentage of cells with positive staining. The staining intensity of FBXO22 was scored 0, 1, 2, or 3 (0, negative; 1, weak; 2, moderate; 3, strong); the percentage of the FBXO22- positive stained cells was graded as 0 (<5%), 1 (5–25%), 2 (25–50%), 3 (50–75%), or 4 (75–100%). The immunoreactive score (IRS) of each section was calculated by multiplying the scores of staining intensity and the percentage of positive cells. On the basis of the IRS, staining patterns were divided into two classes: negative (IRS: 0–6) and positive (IRS: 7–12) expression.

### Cell Lines and Cell Culture

Human epithelial ovarian cancers cell lines (OVCAR3, HO8910, and A2780) and human ovarian epithelium cells (IOSE) were obtained from the Type Culture Collection of the Chinese Academy of Sciences (Shanghai, China). The OVCAR3 cells were cultured in an RPMI 1640 medium supplemented with 20% fetal bovine serum (FBS) and 0.1 mg/ml insulin. The HO8910, A2780, and IOSE cell lines were cultured in an RPMI 1640 medium supplemented with 10% FBS. Then, 100 U/ml streptomycin/penicillin was added to the RPMI 1640 medium. All the cells were cultured in an incubator at 37°C with 5% CO_2_.

### Immunofluorescence

Cells were seeded on coverslips, fixed in 4% paraformaldehyde at room temperature for 15 min, rinsed in PBS for three times, permeabilized in 0.3% Triton X-100 for 15 min, rinsed in PBS for three times, and blocked in 2% bovine serum albumin at room temperature for 1 h. Then, coverslips were incubated with primary antibodies 1:100 diluted in staining buffer (1% bovine serum albumin in 0.3% Triton X-100/PBS) overnight at 4°C in a humid chamber. After washing three times, secondary antibodies (Alexa Fluor 595; Abcam) were applied in a 1:200 dilution in staining buffer for 1 h at 37°C in a humid chamber in the dark. After washing, coverslips were mounted with Vectorshield with 4′,6-diamidino-2-phenylindole (DAPI; Beyotime Biotechnology, Nanjing, China). Fluorescent signals were captured with a confocal laser scanning microscope (ZEISS LSM880, Germany).

### FBXO22 Small Interfering RNA or Overexpression Plasmid Transfection

Small interfering RNA (siRNA) specific for FBXO2 (siFBXO22#1, siFBXO22#2, and siFBXO22#3), autophagy related 5 (ATG5, si-ATG5), and nonspecific control (NC) were purchased from Gene-Pharma (Shanghai, China) and transfected with siLentFect lipid reagent (Bio-Rad Laboratories, Inc.) according to the manufacturer’s protocol when the epithelial ovarian cancers cells grew to 30–40% confluency. Approximately 6 h after transfection, the medium containing transfection reagents was replaced with a fresh medium. The siRNA sequences were as follows:

NC: sense, 5′-UUC​UCC​GAA​CGU​GUC​ACG​UTT-3′;
antisense, 5′-ACG​UGA​CAC​GUU​CGG​AGA​TT-3′.
siFBXO22#1: sense, 5′-GGU​GGG​AGC​CAG​UAA​UUA​UTT-3′;
antisense, 5′-AUA​AUU​ACU​GGC​UCC​CAC​CTT-3′.
siFBXO22#2: sense, 5′-GUU​CGC​AUC​UUA​CCA​CAU​ATT-3′;
antisense, 5′-UAU​GUG​GUA​AGA​UGC​GAA​CTT-3′.
siFBXO22#3: sense, 5′-GCA​CCU​UCG​UGU​UGA​GUA​ATT-3′;
antisense, 5′-UUA​CUC​AAC​ACG​AAG​GUG​CTT-3′.
siATG5: sense, 5′-CAG​UUU​GGC​ACA​AUC​AAU​ATT -3′;
antisense, 5′-UAU​UGA​UUG​UGC​CAA​ACU​GTT-3′.

Overexpression plasmid (HA-FBXO22) and nonspecific control (pcDNA3.1-Ctrl) were purchased from You Bio (Hunan, China) and transfected with Lipo2000 (Invitrogen, Shanghai, China) according to the manufacturer’s protocol when the epithelial ovarian cancers cells grew to 80–90% confluency. Approximately 6 h after transfection, the medium containing transfection reagents was replaced with a fresh medium.

### Plasmid Construction

shFBXO22-LV3, Ctrl-LV3, Flag-FBXO22-LV5, and Ctrl-LV5 lentivirus (Gene-Pharma) were used for the stable suppression and overexpression of FBXO22. After 48 h of infection on HO8910 cells with the lentivirus, stable cells were extracted using 2 μg/ml puromycin for 14 days.

### Antibodies and Western Blot Analysis

This procedure was completed using the following primary antibodies raised against: FBXO22, GAPDH, P62, BECN1 and ATG5 (Proteintech Group); MMP-2, ERK, p-ERK, P38, p-P38, p-90RSK, RAC1 and BCL-2 (Cell Signaling Technology), and LC3 I/II (Abcam). The secondary antibodies were goat anti-rabbit and goat anti-mouse corresponding HRP (Beijing Biodragon Immunotechnologies Co., Ltd., Beijing, China). The protein bands were determined using a Tanon 5200 automated chemiluminescent imaging analysis system with ECL reagents (Tanon, Shanghai, China).

### Cell Proliferation Assay

After knock down or overexpression of FBXO22 in HO8910 and OVCAR3 cells, cell proliferation was monitored by the cell counting kit-8 (CCK-8; Beyotime Biotechnology, Nanjing, China). After knockdown or overexpression of FBXO22 in HO8910 and OVCAR3 cells, 5 × 10^3^ cells were seeded in a 96-well culture plate and then incubated at 37°C in a humidified atmosphere containing 5% CO_2_ for 24 h. Then, 10 μl of CCK-8 solution was added 24, 48, 72, and 96 h after plating, and the cells were incubated at 37°C for another 1 h. Absorbance was determined at 450 nm.

### Wound Healing Assays

In the wound healing assays, cells were planted in six-well plates and cultured to 80% confluence. Then, a sterile 10 μl pipette tip was used in making artificial scratches in each well. Suspended cells were washed away with PBS, then the cells were cultured in a medium with 1% FBS. Cell migration distance was photographed at 0 and 24 h under an inverted light microscope.

### Cell Migration and Invasion Assays

Assays on cell migration and invasion were performed using Transwell filter inserts (8.0 μm pore size with polycarbonate membranes) precoated with Matrigel (BD Biosciences, NJ, United States) and those that were not. The cells underwent serum starvation overnight, and 2 × 10^5^ cells were seeded into the upper chamber for the migration assay kit. In the same chamber, 4 × 10^5^ cells were seeded into a medium without serum for the invasion assay. The lower chamber was filled with RPMI 1640 supplemented with 20% FBS. After 24 and 48 h of incubation at 37°C in 5% CO_2_, the cells were fixed in the membrane with methanol (90%) and then stained with crystal violet. The cells in the upper chamber were removed gently. Afterward, the traversed cells were dried and counted.

### Electron Microscopy

shFBXO22–LV3, Ctrl–LV3, Flag–FBXO22–LV5, and Ctrl–LV5 cells (1 × 10^7^) were pre-fixed in 2.5% glutaraldehyde and 1% osmium tetroxide and then incubated with 1% OsO_4_ for 3 h at 4°C, dehydrated in a graded series of ethanol, and flat-embedded in epoxy resin. Ultrathin sections were stained with uranyl acetate and lead citrate and observed under a transmission electron microscope (Tecnai G2 Spirit Twin/*Tecnai G2 Spirit Twin).

### Quantitation of Autophagy With mRFP-GFP-LC3 Adenovirus

After si-Ctrl, si-FBXO22, pcDNA3.1, and HA-FBXO22–pcDNA3.1 were transfected into the HO8910 cells, mRFP-GFP-LC3 adenoviral particles (50 MOI, Hanbio Biotechnology Co., Ltd. Shanghai, China) were infected for 24 h. Fluorescent signals were captured with a confocal laser scanning microscope (ZEISS LSM880, Germany). The number of autolysosomes and autophagosomes was determined by counting the red puncta or yellow puncta, respectively. Thirty randomly selected cells per experimental group were analyzed.

### Lysosomal Tracker

A lysosomal tracker (Beyotime, Shanghai, China) was diluted with HBSS (1:10,000) and then incubated at 37 °C under a humidified atmosphere containing 5% CO_2_ for 1 h. Fluorescent signals were captured with a confocal laser scanning microscope (ZEISS LSM880, Germany).

### Xenograft Mice Model *In Vivo*

Eight six-week-old BALB/c nude mice were used. Stable HO8910 cell lines constructed with Ctrl-LV3 and shFBXO22-LV3 were injected into the left and right backs successively with the number of 5 × 10^6^. Five weeks after injection, the mice were sacrificed, and their subcutaneous tumors were excised and fixed in 10% buffered formalin for the detection of FBXO22 and Ki67expression levels through IHC analysis.

### Metastatic Mice Model *In Vivo*

Sixteen six-week-old BALB/c nude mice were divided into two groups and injected i.p., with Ctrl-LV3 and shFBXO22-LV3 cells (5 × 10^6^) respectively. Fourty days after injection, the mice were sacrificed, and their organs including colon, lung, kidney, liver, spleen and heart were excised and made into paraffin sections for analysis of metastasis by HE staining.

### Statistical Analysis

Quantitative data are expressed as the means ± SD of at least three independent experiments. All experimental values were evaluated using GraphPad Prism 8.3.0 (GraphPad software). Differences between two groups were analyzed with Student’s t test, whereas differences among more than two groups were evaluated by one-way ANOVA for independent samples or ANOVA for repeated measurements followed by Tukey post-hoc test. In all cases, *p* < 0.05 was considered statistically significant.

## Results

### The Expression of FBXO22 Is Associated With Clinical Pathological Factors in Epithelial Ovarian Cancers

**FIGURE 1:**
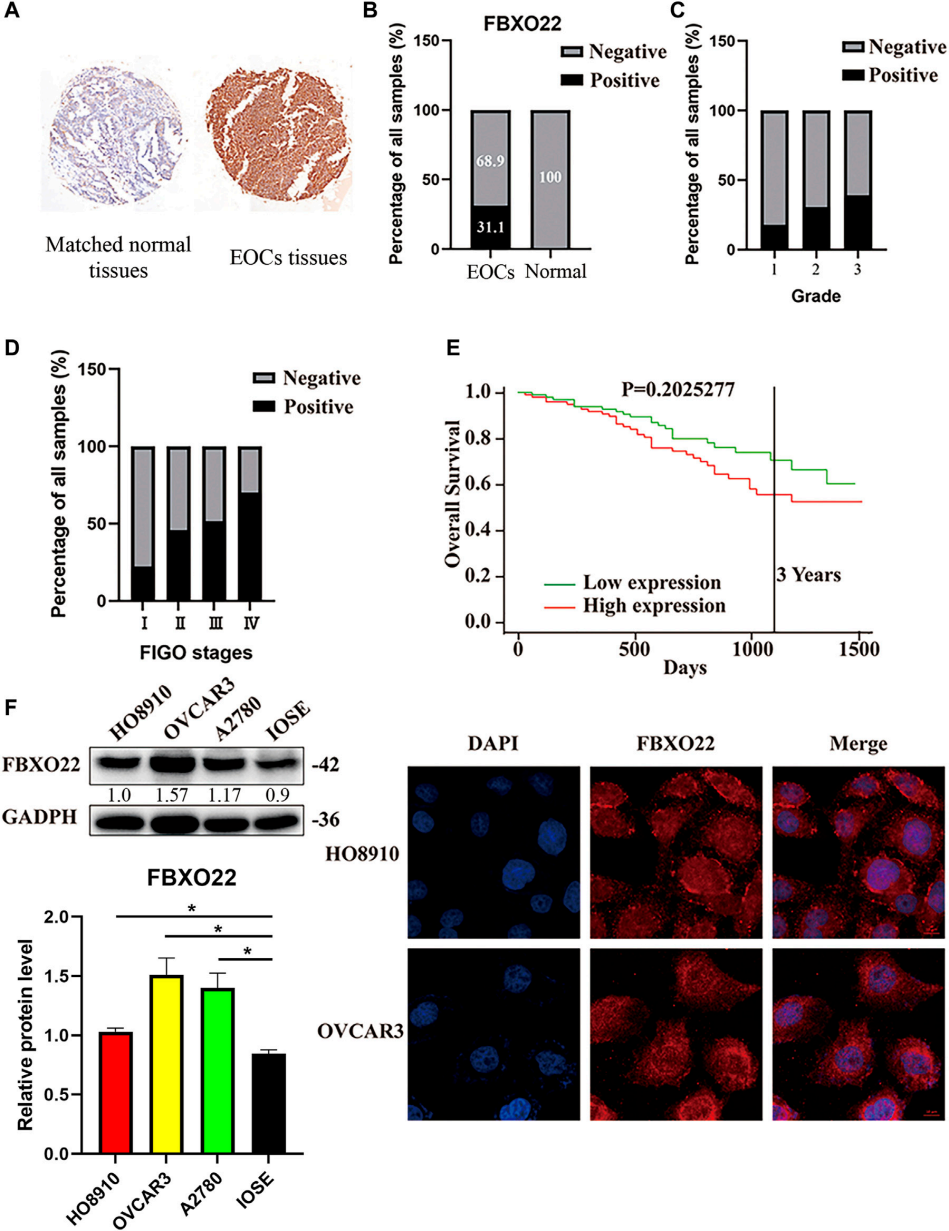
The high expression of FBXO22 is associated with clinical pathological factors in EOCs. **(A)** IHC staining of FBXO22 in TMA containing 251 EOCs tissues and 10 matched normal tissues (×20). **(B)** Analysis of FBXO22 expression in EOCs and matched normal tissues. **(C)** Analysis between FBXO22 expression and pathological grade. **(D)** Analysis between FBXO22 expression and FIGO stages. **(E)** Survival analysis of ovarian cancers in TCGA. **(F)** Western blot results of FBXO22 expression in epithelial ovarian cancers cell lines (HO8910, OVCAR3, A2780) and ovarian epithelial cells (IOSE). Independent samples *t*-test was used for statistics and the results were expressed as mean ± SD, *, *p* < 0.05; ***, *p* < 0.001 (*N* = 3). **(G)** Immunofluorescence of FBXO22 in HO8910 and OVCAR3 cells (×100).

To evaluate whether the FBXO22 protein expression is related to EOCs, we performed immunohistochemistry experiments across an entire TMA containing 251 EOCs tissues and 10 matched normal tissues. The resulting FBXO22 staining intensity was quantified using IRS. Samples with IRS in the range of 0–6 or 7–12 were classified as negative or positive FBXO22 expression, respectively (Figure 1A). The statistic results show that negative or positive FBXO22 expression accounted for 68.9% or 31.1% of the 251 tumor samples analyzed, respectively (Figure 1B). The expression of FBXO22 in the all 10 matched normal tissues were negative (Figure 1B).

Moreover, the FBXO22 expression was positively correlated with clinical FIGO stages (*p* < 0.001) and pathological grades (*p* = 0.015) (Figures 1C,D; Table 1). No significant difference between FBXO22 expression and age was observed (Table 1, *p* = 0.662). Analysis of the epithelial ovarian cancer patients in TCGA shown that there was no significant difference in survival (Figure 1E, *p* = 0.203). These results shown that EOCs patients have higher expression of FBXO22 and the expression of FBXO22 is associated with clinical pathological factors.

**TABLE 1:** Expression of FBXO22 and clinicopathological factors of ovarian cancer patients.

Features	Total	Expression of FBXO22		*χ* ^2^ value	*p* Value
		Negative (%)	Positive (%)		
Age (years)				0.191	0.662
<49	121	85 (70.2)	36 (29.8)		
≥49	130	88 (67.7)	42 (32.3)		
FIGO stages				22.993	<0.001
I	175	136 (77.7)	39 (22.3)		
II	35	19 (54.3)	16 (45.7)		
III	31	15 (48.4)	16 (51.6)		
IV	10	3 (30.0)	7 (70.0)		
Grade				8.464	0.015
1	62	51 (82.3)	11 (17.7)		
2	79	55 (69.6)	24 (30.4)		
3	110	67 (60.9)	43 (39.1)		

Consistently, increased FBXO22 expression was also detected in three EOC cell lines (HO8910, OVCAR3 and A2780) as compared with normal ovarian epithelial cell line IOSE (Figure 1F). Moreover, the subcellular location of FBXO22 in HO8910 and OVCAR3 cells was determined by immunofluorescence, showing that FBXO22 was both nuclear and cytoplasmic (Figure 1G).

### FBXO22 Promotes the Growth of Epithelial Ovarian Cancers *In Vitro* and *In Vivo*

**FIGURE 2:**
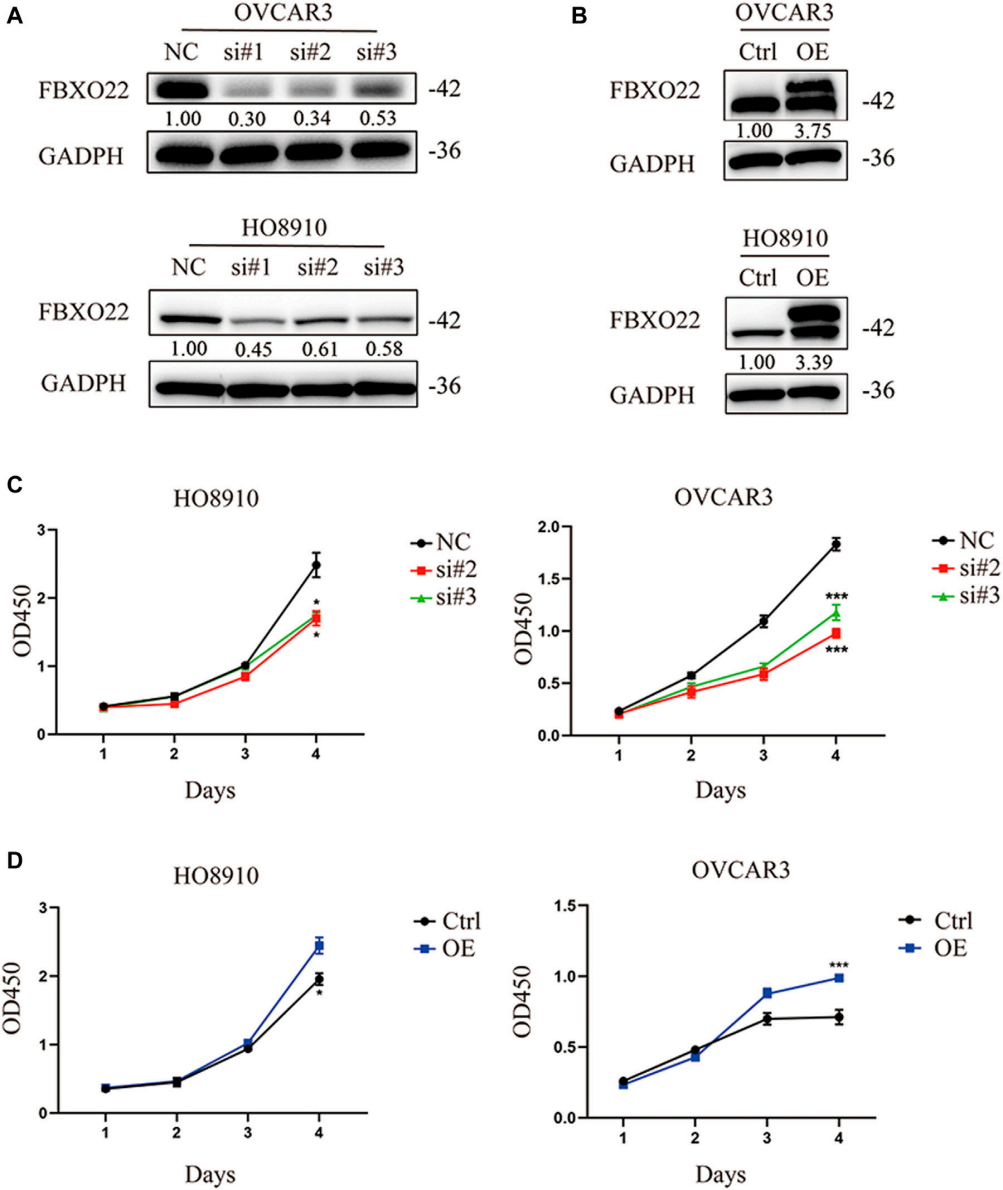
FBXO22 promotes the growth of EOCs *in vitro*. **(A)** Knockdown of FBXO22 were confirmed at the protein level in OVCAR3 and HO8910 cells by western blot. **(B)** Overexpression of FBXO22 were confirmed at the protein level in OVCAR3 and HO8910 cells by western blot. **(C)** Cell proliferation of FBXO22 knockdown HO8910 and OVCAR3 cells by CCK8 assay. **(D)** Cell proliferation of FBXO22 overexpression HO8910 and OVCAR3 cells by CCK8 assay. Independent samples *t*-test was used for statistics and the results were expressed as mean ± SD, *, *p* < 0.05; **, *p* < 0.01; ***, *p* < 0.001 (*N* = 6).

To investigate the biological function of FBXO22 in epithelial ovarian cancers cell proliferation, we transfected HO8910 and OVCAR3 cells with negative control siRNA (NC) and siRNAs targeting FBXO22 (si#1, si#2 and si#3) successively (Figure 2A). Then, CCK-8 cell proliferation assays were performed. The knockdown of FBXO22 inhibited the growth of epithelial ovarian cancers cells (Figure 2C). Conversely, the proliferation of epithelial ovarian cancers cells was drastically increased in both cell lines overexpressing FBXO22 compared with the control groups (Figures 2B,D).

**FIGURE 3:**
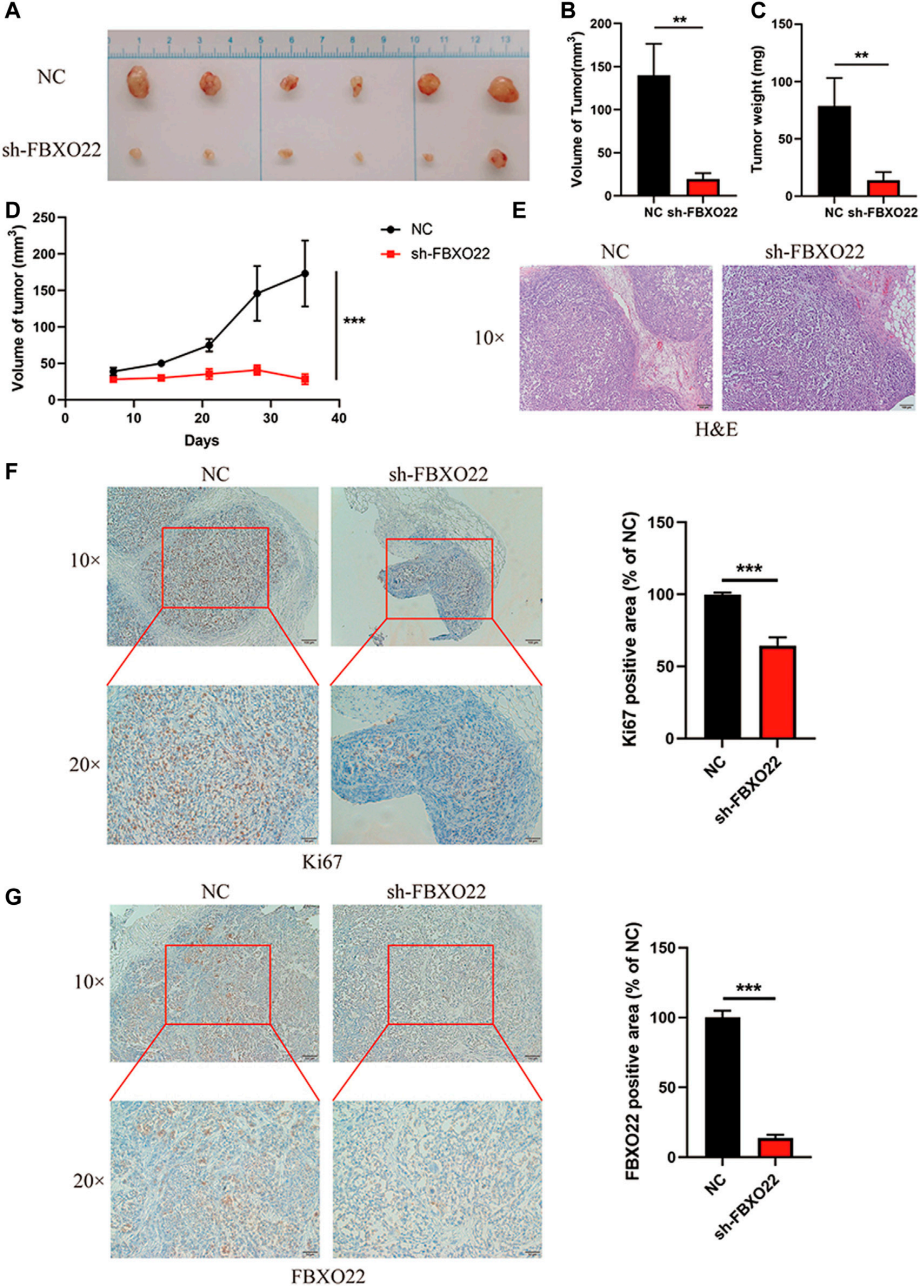
FBXO22 promotes the growth of EOCs *in vivo*. **(A)** Images of subcutaneous tumors resected from the mice after 5 weeks of growth *in vivo*. **(B–D)** Analysis of volume and weight of tumors. Data were presented as mean ± SD, **, *p* < 0.01; ***, *p* < 0.001 (*N* = 6). **(E)** HE staining of the subcutaneous tumors. **(F,G)** Immunostaining of KI67 and FBXO22 were performed in the subcutaneous tumors (×10 and ×20). Independent samples *t*-test was used for statistics and the results were expressed as mean ± SD, ***, *p* < 0.001 (*N* = 6).

Furthermore, we established Ctrl-LV3 (NC) and shFBXO22-LV3 (sh-FBXO22) stable cell lines to verify the function of FBXO22 in the regulation of epithelial ovarian cancers cell *in vivo*. The cells were subcutaneously injected into BALB/c nude mice. The mice were sacrificed 5 weeks after the implementation, and the tumors were exposed (Figure 3A). Differences in tumor volume and weight between the two groups were statistically analyzed (Figures 3B–D). The results shown that knockdown of FBXO22 inhibited the growth of epithelial ovarian cancers *in vivo*. HE and IHC staining indicated that FBXO22 deficiency reduced the growth of epithelial ovarian cancers (Figures 3E–G). The results revealed that FBXO22 promotes cell proliferation in epithelial ovarian cancers.

### FBXO22 Promoted Metastasis of Epithelial Ovarian Cancers *In Vitro* and *In Vivo*

**FIGURE 4:**
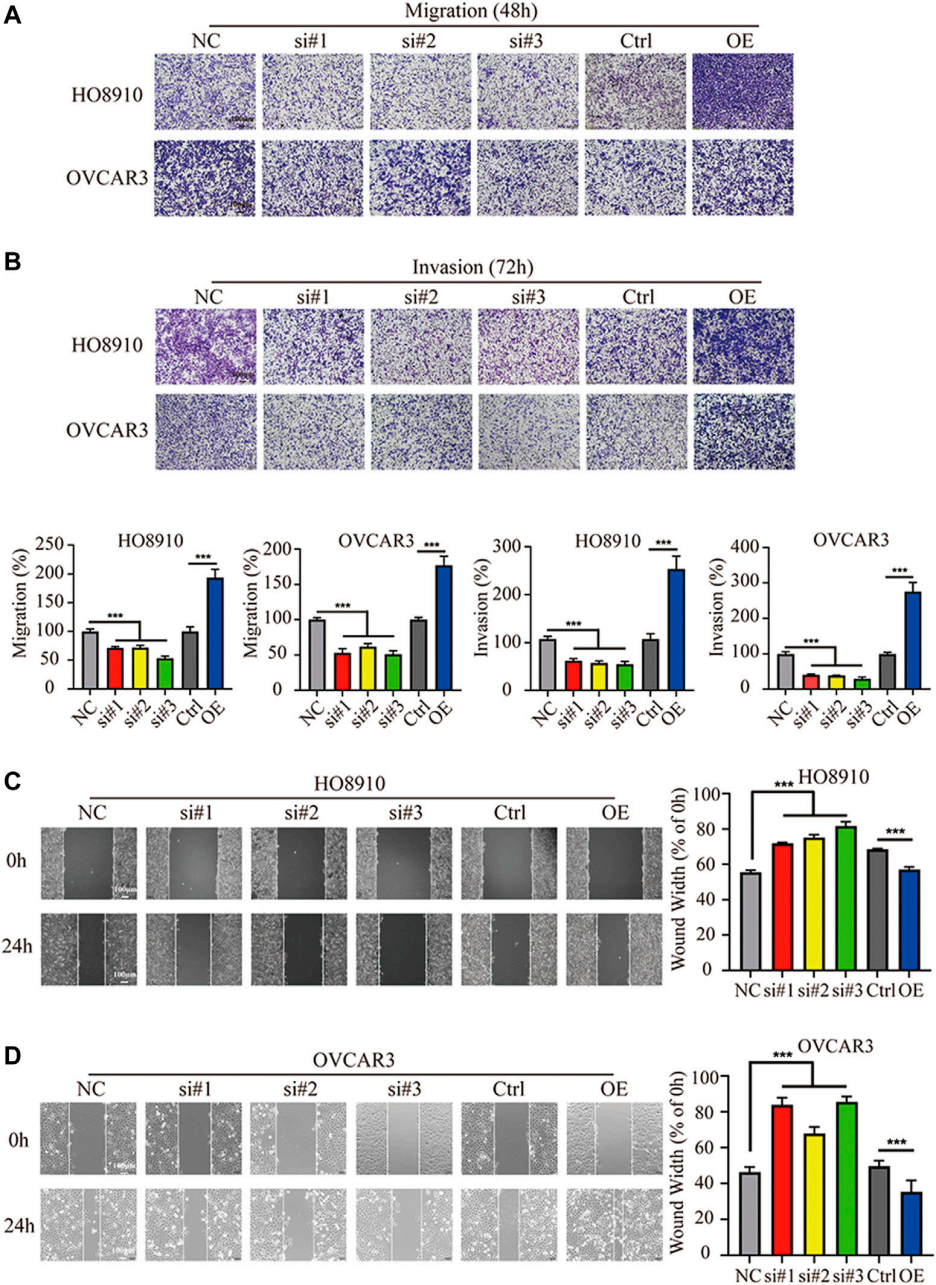
FBXO22 promotes the metastasis of EOCs cells *in vitro*. **(A,B)** Cell wound healing assays in HO8910 and OVCAR3 cells after FBXO22 after treatment with siFBXO22 or overexpression plasmid (×10). Independent sample *t* test was used for statistics between Ctrl and OE, One-way ANOVA was used for NC vs si#1/si#2/si#3, the statistical results were expressed as mean ± standard deviation, ***, *p* < 0.001 (*N* = 5). **(C,D)** The knockdown of FBXO22 inhibits while the overexpression of FBXO22 promotes the migration and invasion of epithelial ovarian cancers cells (×10). Independent sample *t* test was used for statistics between Ctrl and OE, One-way ANOVA was used for NC vs si#1/si#2/si#3, the statistical results were expressed as mean ± SD, ***, *p* < 0.001 (N = 5).

The effect of FBXO22 on migration and invasion of EOCs were also explored. Knockdown of FBXO22 significantly inhibited the migration ability of HO8910 and OVCAR3 cells when compared with control cells (Figure 4A), as evidenced by wounding healing assay (Figures 4C,D). In contrast, FBXO22 overexpression promoted the migration ability of HO8910 and OVCAR3 cells (Figures 4A,C,D). In addition, Transwell matrige invasion assays also shown that FBXO22 knockdown reduced the invasion ability and overexpression FBXO22 enhanced the invasion ability, respectively (Figure 4B).

**FIGURE 5:**
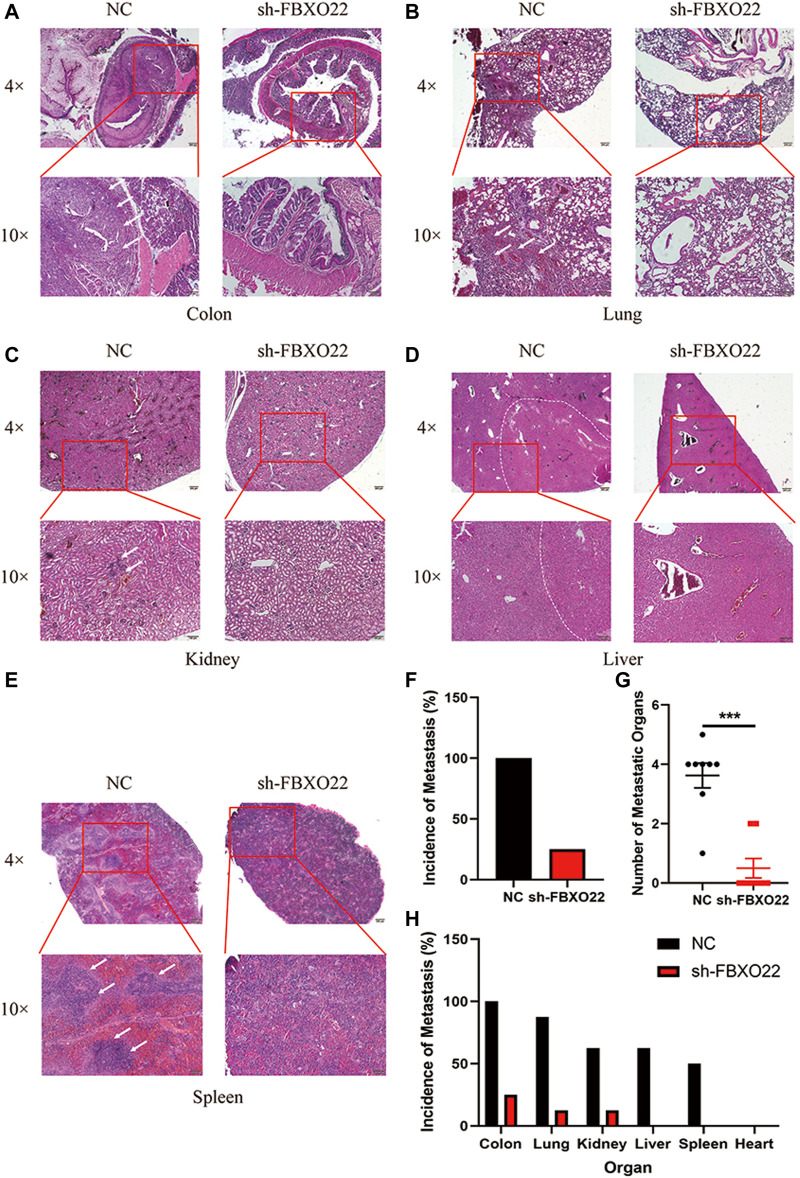
FBXO22 promotes the metastasis of EOCs *in vivo*. HE staining of the NC and sh-FBXO22 mice organs: **(A)** colon; **(B)** lung; **(C)** kidney; **(D)** liver; **(E)** spleen; **(F)** Analysis of metastasis incidence in NC and sh-FBXO22 mice; **(G)** Analysis of metastatic organs number; **(H)** Analysis of metastasis incidence in organs. Independent samples *t*-test was used for statistics and the results were expressed as mean ± SD, ***, *p* < 0.001 (*N* = 6).

To further detect the role of FBXO22 in the peritoneal metastasis of EOCs, we used an intraperitoneal injection mice model. In brief, 2 × 10^6^ HO8910 NC/sh-FBXO22 cells were injected to 7-week-old nude mice, and the peritoneal metastasis status was analyzed 8 weeks later. Compared with the NC group, sh-FBXO22 cells formed fewer metastatic foci in colon, lung, kidney, liver and spleen (Figures 5A–E). We analyze the incidence of metastasis (Figure 5F) and the number of metastatic organs (Figure 5G), as well as the incidence of metastasis in every organ (Figure 5H).

In conclusion, our results shown that FBXO22 promoted metastasis of EOCs both *in vitro* and *in vivo*.

### FBXO22 Promotes the Growth and Metastasis of Epithelial Ovarian Cancers Cells *via* the MMP2 and Mitogen-Activated Protein Kinase Pathways

**FIGURE 6:**
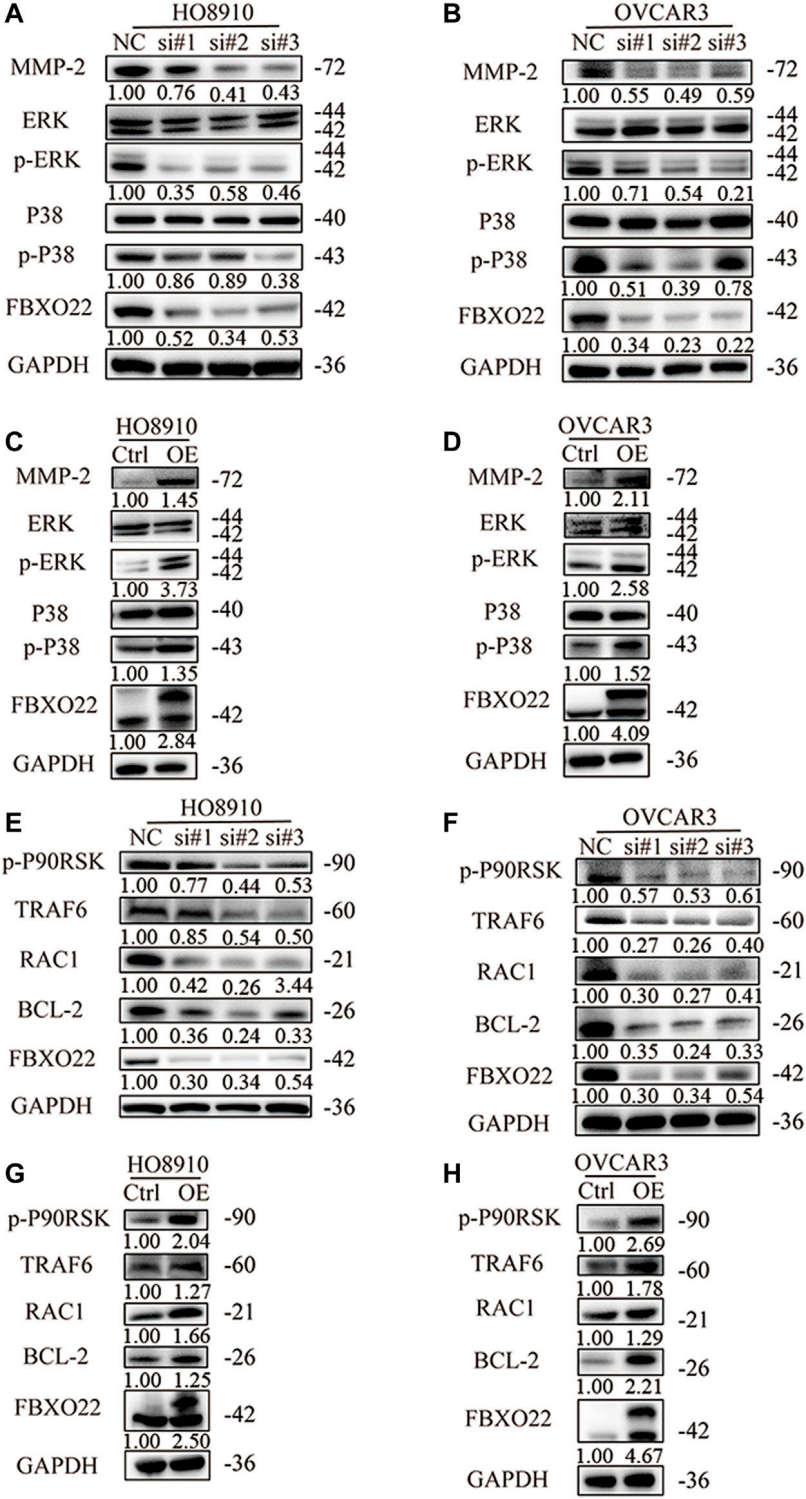
FBXO22 promotes the growth and metastasis of EOCs cells via MMP2 and MAPK pathway. Western blot analysis was performed to measure the expression of MMP-2 and MAPK pathway related proteins in HO8910 and OVCAR3 cells after treatment with siFBXO22 **(A)**, and those in FBXO22 overexpressed HO8910 and OVCAR3 cells **(B)**. Western blot analysis was performed to measure the expression of TRAF6, RAC1, p-P90RSK and BCL-2 in FBXO22 interfered HO8910 and OVCAR3 cells **(C)**, and those in FBXO22 overexpressed HO8910 and OVCAR3 cells **(D)**.

To explore the mechanism by which FBXO22 promotes the metastasis of epithelial ovarian cancers cells, we identified some related pathways and proteins. Matrix metalloproteinases (MMPs), as important enzymes that degrade the extracellular matrix, play an important role in mediating tumor angiogenesis, metastasis, and invasion. MMP-2 is an important MMP (Malemud, 2006). We detected the content of MMP-2 in epithelial ovarian cancers cells with interfered and overexpressed FBXO22. The results shown that MMP2 expression was decreased after interfering with FBXO22 and increased after the overexpression of FBXO22 (Figures 6A,B).

MAPKs constitute a group of serine threonine protein kinases that can be activated by a series of extracellular stimulus signals and mediate signal transduction from the cell membrane to the nucleus. They regulate many physiological activities, such as inflammation, apoptosis, carcinogenesis, invasion, and metastasis, particularly those of tumor cells. ERK1/2 and P38 are the two important members of MAPK (Fan and Chambers, 2001). We detected the levels of ERK1/2, P38, p-ERK1/2, and p-P38. The results shown that FBXO22 can promote the activation of ERK1/2 and P38 (Figures 6A,B). Subsequently, we verified some relevant proteins. Tumor necrosis factor receptor associated protein 6 (TRAF6) was reported to regulate MAPK pathway (Liu et al., 2017; Rasheed et al., 2019; Ahmedy et al., 2020). We found that the expression levels of TRAF6, as well as downstream proteins RAC1, p-P90RSK and BCL-2 changed respectively (Figures 6C,D). In conclusion, FBXO22 promotes the growth and metastasis of epithelial ovarian cancers cells via the MMP-2 and MAPK pathways.

### FBXO22 Inhibits the Autophagy of Epithelial Ovarian Cancers

**FIGURE 7:**
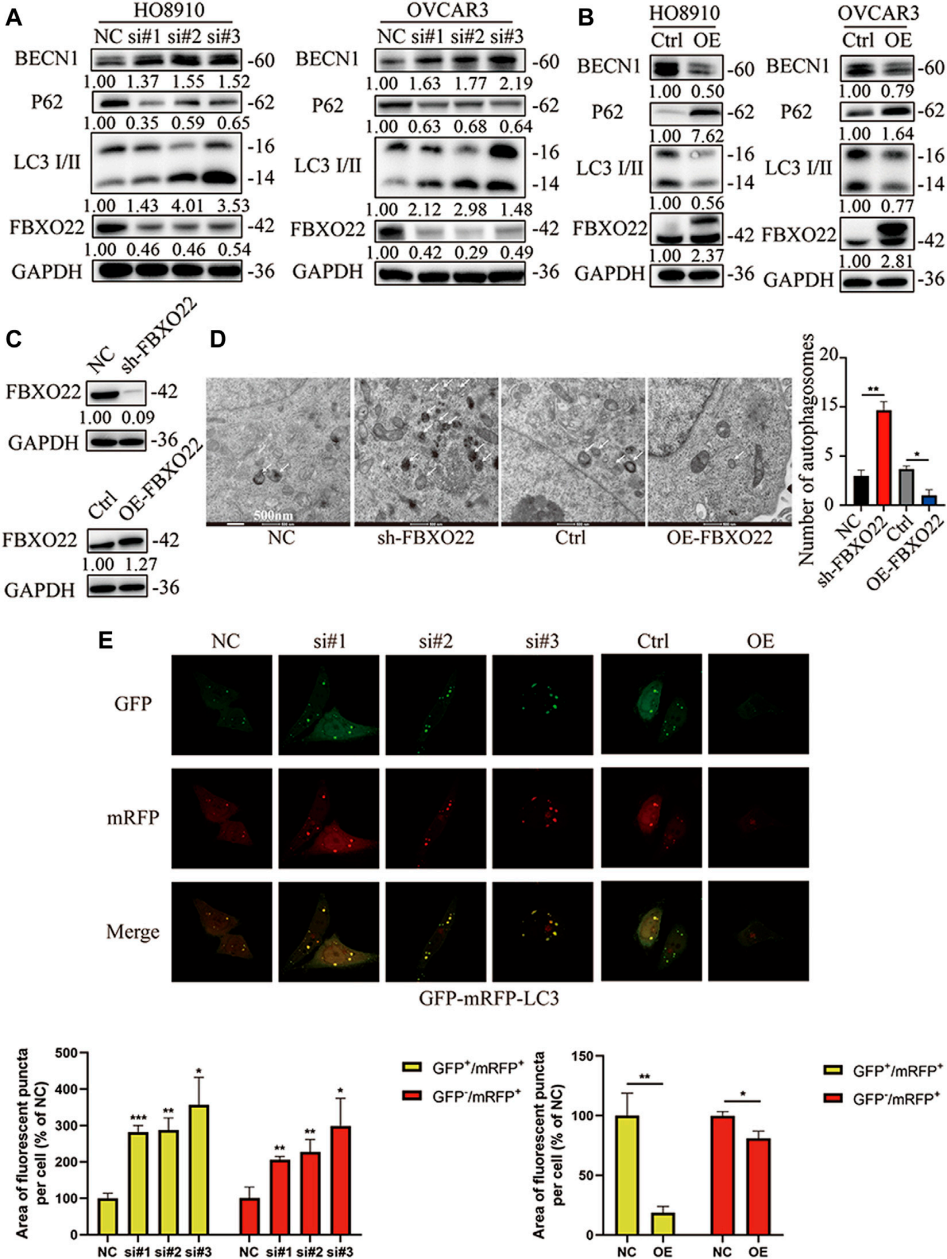
FBXO22 inhibits autophagy of EOCs cells. Western blot shows the expression of P62 and LC3 I/II proteins in FBXO22 interfered HO8910 and OVCAR3 cells **(A)**, and those in FBXO22 overexpressed HO8910 and OVCAR3 cells **(B)**. s.t: short time exposure, l.t: long time exposure; **(C)** Expression of FBXO22 in stable cell lines was confirmed by western blot. **(D)** Autophagosomes observed by electron microscopy (×8,000). **(E)** mRFP-GFP-LC3 shows the change of autophagy flux in HO8910 cells by confocal (×100). Independent samples *t*-test was used for statistics and the results were expressed as mean ± SD, *, *p* < 0.05; **, *p* < 0.01; ***, *p* < 0.001 (*N* = 5).

Significantly, TRAF6 and BCL-2, which were proved correlated with FBXO22 in former study, reported to regulate autophagy (Shi and Kehrl, 2010; Fernández et al., 2018; Min et al., 2018). Thus, we hypothesized that FBXO22 could affect autophagy. LC3 I/II is one of the most important autophagy markers. The transformation from LC3 I to LC3 II reflects the process of autophagy, and P62 is the substrate of autophagy. The results shown that the rate of transformation from LC3 I to LC3 II increased and the substrate P62 decreased after FBXO22 interference, indicating that autophagy was enhanced (Figure 7A). By contrast, after the overexpression of FBXO22, the rate of transformation from LC3 I to LC3 II decreased, and the substrate p62 increased, indicating that autophagy was reduced (Figure 7B). BECN1, which plays a central role in autophagy, increased after FBXO22 knockout and decreased after FBXO22 overexpression (Figures 7A,B).

Then, stable cell lines were established (Figure 7C). Autophagosomes were observed through electron microscope, and the results confirmed that FBXO22 inhibits the autophagy flux of epithelial ovarian cancers cells (Figure 7D). To observe the autophagy flux directly, we infected the cells with the autophagy dual fluorescent virus mRFP-GFP-LC3. The results shown that FBXO22 knockdown increased the number of yellow autophagosomes, indicating that the induced production of autophagosomes increased. Meanwhile, the number of produced autophagosomes was reduced after FBXO22 was overexpressed (Figure 7E).

### The Regulation of Autophagy by FBXO22 Depends on p-ERK

**FIGURE 8:**
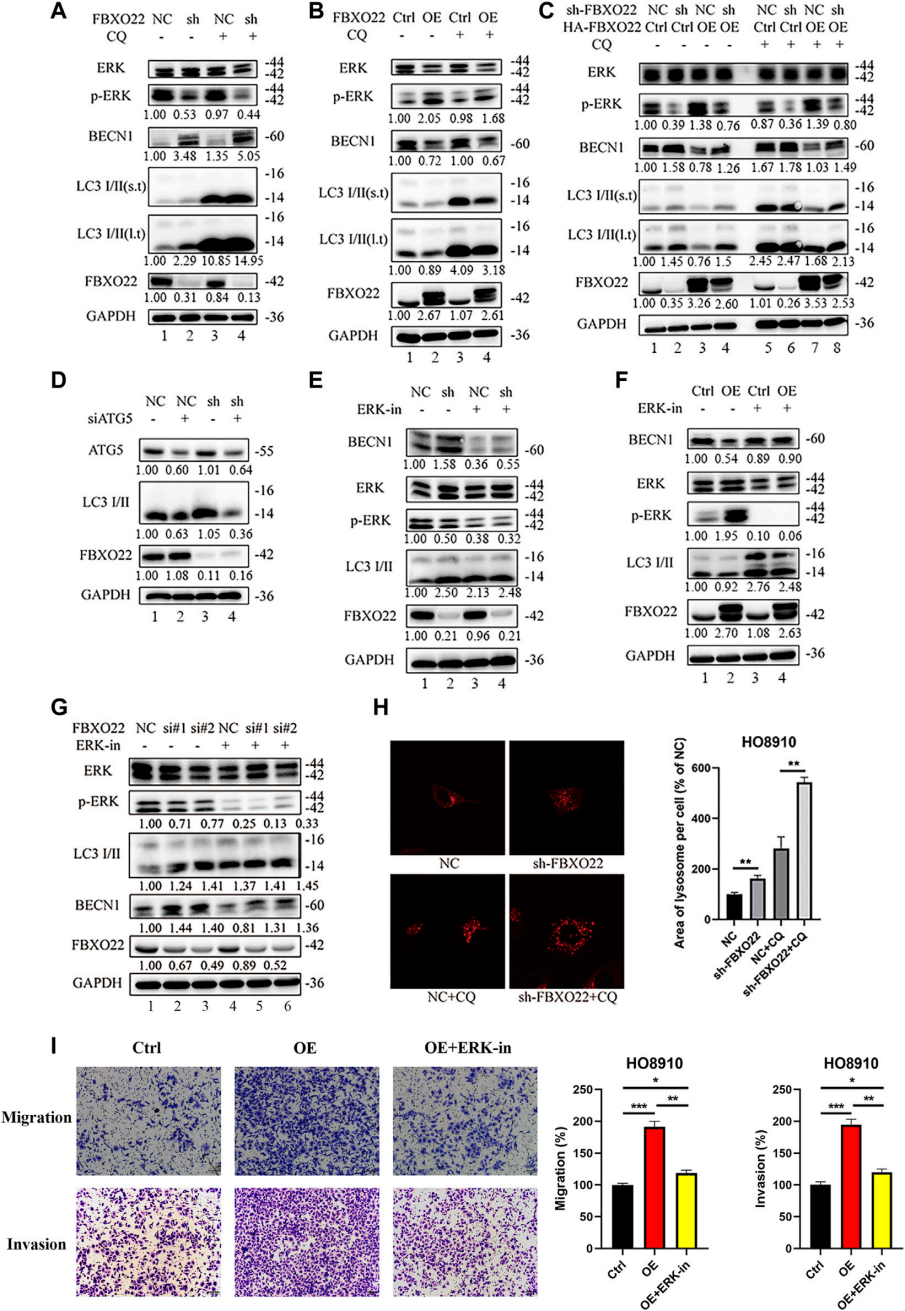
The inhibition in autophagy of FBXO22 is p-ERK depended. **(A)** Western blot results of ERK, p-ERK, BECN1, LC3 I/II, FBXO22 and GAPDH in the stable NC and sh-FBXO22 HO8910 cells. **(B)** HO8910 cell were transfected with pcDNA3.1-Ctrl (Ctrl) or HA-FBXO22 plasmid (OE), then treated with CQ. **(C)** The stable NC and sh-FBXO22 HO8910 cells were transfected with pcDNA3.1-Ctrl (Ctrl) or HA-FBXO22 plasmid (OE), then treated with CQ. **(D)** The stable NC and sh-FBXO22 HO8910 cells were transfected with NC and siATG5. **(E)** The stable NC and sh-FBXO22 HO8910 cells were treated with ERK inhibitor. **(F)** HO8910 cell were transfected with pcDNA3.1-Ctrl (Ctrl) or HA-FBXO22 plasmid (OE), then treated with ERK inhibitor. **(G)** HO8910 cell were transfected with Negative Control siRNA (NC) or siRNAs of FBXO22 (si#1, si#2 and si#3), then treated with ERK inhibitor. Protein levels were demonstrated by Western blot. **(H)** The lysosomal tracker was transfected in the stable NC and sh-FBXO22 HO8910 cells. **(I)** Migration and invasion assay in HO8910 cells treated with Ctrl plasmid **(left)**, OE plasmid **(middle)** and OE plasmid as well as ERK1/2 inhibitor **(right)**. Independent samples *t*-test was used for statistics and the results were expressed as mean ± SD, *, *p* < 0.05; **, *p* < 0.01; ***, *p* < 0.001 (*N* = 5).

Chloroquine (CQ), an inhibitor of LC3B degradation in autolysosomes, was used to verify autophagy flux. Autophagy flux increased after FBXO22 knockout but decreased after FBXO22 overexpression, and was negatively correlated with p-ERK (Figures 8A,B lane 1 and 2). The result was validated again after CQ was added that FBXO22 inhibits the p-ERK correlated autophagy flux (Figures 8A,B lane 3 and 4). Furthermore, the increase in autophagy flux mediated by FBXO22 knockout was suppressed after FBXO22 was re-overexpressed (Figure 8C lane 3,4 vs. 1,2). The result was validated again after CQ was added (Figure 8C lane 7,8). ATG5 involved in autophagic vesicle formation and is a key protein in the process of autophagosome production. We then inhibited the formation of autophagosomes by knocking down ATG5 with siRNA and found that the increased autophagy flux was suppressed (Figure 8D).

To validate the essential functions of p-ERK, ERK1/2 inhibitor 1, a potent orally bioavailable ERK1/2 inhibitor, was used. The results shown that the level of p-ERK was decreased by the ERK inhibitor, which enhanced autophagy flux in HO8910 cells with FBXO22 knockdown or overexpression (Figures 8E–G). Moreover, lysosomal tracker was used. FBXO22 knockout increased the lysosomes (Figure 8H). In addition, the rescue Transwell matrige migration and invasion assays were carried out. As we can see, the migration and invasion percentage were declined in FBXO22 overexpression cells after using ERK1/2 inhibitor (Figure 8I).

In conclusion, FBXO22 inhibits the autophagy of EOCs, and the inhibition function is p-ERK dependent.

## Discussion

There is tremendous amount of research and focus on EOC as it presents in advanced stages and is the most fatal of the gynecologic malignancies. Since EOC does not have symptoms specific to cancer, there are no early screening and detection modalities (Kim et al., 2012; Lokadasan et al., 2016). Thus, around 75% of women are diagnosed in advanced stage disease (FIGO IIIc or IV) (Kim et al., 2012; Lokadasan et al., 2016). Moreover, clinical treatments for epithelial ovarian cancers have bottlenecks, and the molecular targets of epithelial ovarian cancers are extremely difficult to find, and thus the molecular mechanism of epithelial ovarian cancers remains a major research topic. Our study found that FBXO22 expression increased, and the increase was correlated with clinicopathological factors in patients with epithelial ovarian cancers. These results shown that FBXO22 has an inducing effect *in vivo* and *in vitro*, showing that FBXO22 is a potential target for the clinical diagnosis and treatment of epithelial ovarian cancers.

E3 ligase is the core component of the ubiquitination cascade. It controls the specificity of a substrate and directly binds to the substrate. Hundreds of E3 ubiquitin ligases are found in humans (Skaar et al., 2013). Among them, SKP1-cullin 1 (CUL1)-F-box (SCF) E3 ligase complex is by far the most characteristic E3 ligase family. The SCF complex is composed of four subunits, namely, the adaptor protein SKP1, the ring finger protein RBX1/2, the scaffold protein CUL1, and the variable F-box protein that recognizes a specific substrate (Frescas and Pagano, 2008). Although not all F-box proteins have good characteristics, many F-box proteins, such as SKP2, FBXW7, FBXO4, and FBXO32, are related to the cancer development and progression and cancer cachexia (Sukari et al., 2016). FBXO22, as a member of the F-box protein family, targets and degrades different proteins, such as PTEN, LKB1, P21, and HDM2. Our research shown that FBXO22 promotes growth and metastasis and inhibits autophagy via the MAPK/ERK pathway. However, the molecule that binds to FBXO22 directly is still unknown.

The results obtained generally supported the antitumor effect of autophagy in the early stage of tumorigenesis. Autophagy facilitates the removal of abnormal and damaged structures or harmful substances from normal cells. If not removed, these structures and substances can promote mutations and other cancer features. Some studies shown that BECN1 is a haploid-deficient tumor suppressor gene and supported the theory that autophagy can inhibit tumor formation as a tumor suppressor gene. A pioneering study of autophagy depletion by knocking out BECN1 (encoding Beclin-1) shown that spontaneous tumors occur in BECN1^+/−^ mice (Aita et al., 1999; Liang et al., 1999; Qu et al., 2003; Yue et al., 2003; Shen et al., 2008).

Some core proteins engage in crosstalk between apoptosis and autophagy, including BCL-2 family members. Anti-apoptotic BCL-2 members inhibit autophagy by binding to BECN1, which contains a functional BH3 domain that inserts into the hydrophobic groove of anti-apoptotic BCL-2 members to regulate autophagy (Kang et al., 2011). Post-translational modifications of BECN1 or BCL-2 contribute to their interaction and autophagy induction. Thr108 phosphorylation of BECN1 promotes BCL-2–BECN1 interaction (Maejima et al., 2013). Anti-apoptotic BCL-2 members are globular proteins formed by nine α-helices with a hydrophobic cleft known as BH3 binding-groove at the surface; this cleft can accommodate the BH3 domains of pro-apoptotic or BH3-only members (Czabotar et al., 2014). Thus, anti-apoptotic BCL-2 members can interact with monomeric BAX or BAK to prevent their oligomerization or to antagonize BH3-only members by interacting with their BH3 domains (Willis et al., 2005; Llambi et al., 2011). Our results shown that FBXO22 increases the expression of BCL-2 and decreases the expression of BECN1. However, whether FBXO22 inhibits the apoptosis in epithelial ovarian cancers remains unclear.

Epithelial ovarian cancer is recognized as a heterogeneous disease and is classified according to histologic subtype: high-grade serous, low-grade serous, clear cell, endometrioid, and mucinous (Gilks and Prat, 2009). The mitogen-activated protein kinase pathway plays a prominent role in the pathogenesis of low-grade serous carcinoma of the ovary, and provides an attractive target for novel therapeutic agents. Mutations in KRAS or BRAF have been reported; the rates vary across studies (KRAS 19–35% and BRAF 2–33%) (Singer et al., 2003; Wong et al., 2010). Downstream inhibition of the MAPK pathway is therefore an attractive target for novel therapeutic agents in low-grade serous carcinoma. Selumetinib, a MEK1/2 inhibitor, demonstrates promising efficacy in women with relapsed low-grade serous carcinoma, and further trials of MEK-inhibition are underway (McLachlan et al., 2016). Our study demonstrated the correlation between FBXO22 and MAPK/ERK, which may provide new ideas for the treatment of epithelial ovarian cancer. However, the deep connection and therapeutic efficacy of combination therapy between FBXO22 and ERK/MEK inhibitor remain to be explored.

In summary, our study is the first to report that FBXO22 promotes growth and metastasis and inhibits autophagy in epithelial ovarian cancers, and these functions depend on the MAPK/ERK pathway. These findings suggest FBXO22 as a novel target of epithelial ovarian cancers assessment and treatment.

## Data Availability

The original contributions presented in the study are included in the article/Supplementary Material, further inquiries can be directed to the corresponding authors.

## Abbreviations

ATG5, autophagy related 5; Bcl-2, B cell lymphoma-2; BECN1, Beclin 1; BH3, BCL-2 homology 3; CQ, chloroquine; EOCs, epithelial ovarian cancers; FBXO22, F-box only protein 22; JNK, c-Jun-NH2-terminal kinases; MAPK, the mitogen-activated protein kinase; MMP-9, matrix metalloproteinase-9; MMPs, matrix metalloproteinases; NC, nonspecific control; ROS, reactive oxygen species; siRNA, small interfering RNA; TMA, tissue microarray; TRAF6, tumor necrosis factor receptor associated protein 6; Vps34, vacuolar protein sorting 34.
